# Conversion of waste cooking oils into environmentally friendly biodiesel

**DOI:** 10.1186/2193-1801-4-S2-P7

**Published:** 2015-11-27

**Authors:** Heidi Lai-ling Li, Peter Hoi-fu Yu

**Affiliations:** School of Biological Sciences, The University of Hong Kong, Hong Kong; Faculty of Science and Technology, Technological and Higher Education Institute of Hong Kong, Hong Kong

## Background

There has been an alarming increase in the dumping of Hong Kong's municipal solid waste (MSW), predominantly food waste, in landfill (9,547 tonnes of MSW per day in 2013) [1]. One way of promoting an environmentally friendly method of minimising food waste is to turn the waste into energy by producing biodiesel from waste cooking oils. Biodiesel is a biodegradable fuel that can be manufactured from food waste with a low price and sustainable supply [2]. It is considered to be a 'green' replacement for fossil fuels because biodiesel is renewable and it reduces carbon emissions to the atmosphere [3]. Biodiesel's main advantages are renewability, biodegradability, non-toxicity, lack of contribution to the greenhouse effect, and safety [4].

In 2013, the EPD (Environmental Protection Department) recorded 16,199 tonnes of used waste oil exported from Hong Kong [5]. We can see a potential market for biodiesel in Hong Kong and the development of biodiesel also brings side benefits to the local market. In recent years, people have been concerned about the problem of carcinogenic gutter oil [6]. Using waste cooking oil to produce biodiesel provides a suitable profitable way to eliminate the raw materials of gutter oil.

The purpose of this research is to produce biodiesel from waste cooking oils in the laboratory and compare the quantity and quality of products made from domestic waste cooking oil, restaurant cooking oil and fresh cooking oil. The feedstocks used were domestic deep-frying canola oil, domestic lard, deep-frying oil obtained from a restaurant and fresh canola oil.

## Methods

The biodiesel was produced by alkali transesterification of the waste oils with the addition of sodium hydroxide and methanol to form methyl esters. The products were subjected to a product yield test, density test, pour point test, cloud point determination and gas chromatography.

## Results

In a comparison of biodiesel yield and quality, household waste cooking oil was a better feedstock than restaurant waste cooking oil.

## Conclusions

Waste cooking oils should be used for biodiesel production to turn waste into energy. Given the required technology and the huge amount of waste cooking oil generated in Hong Kong, mass production of biodiesel is feasible. Domestic waste cooking oil produced biodiesel with a higher yield and better quality profile than biodiesel from restaurant waste cooking oil. Less downstream processing is therefore needed for the mass production of biodiesel from domestic oil and its potential for industrial biodiesel production is higher.

We recommend research into the collection of waste cooking oil at the household level, such as a pilot-scaled collection and production scheme in large estates. The government should provide outlets for biodiesel in Hong Kong, for instance electricity in estates or a subsidy for drivers choosing to use environmentally friendly biodiesel as fuel.
